# An Integrated Analysis of Lactation-Related miRNA and mRNA Expression Profiles in Donkey Mammary Glands

**DOI:** 10.3390/genes13091637

**Published:** 2022-09-12

**Authors:** Yaqi Fei, Yedan Gai, Qingchao Liao, Linxi Zhang, Zheng Li, Bojiang Li, Man Bai, Na Li, Liang Deng

**Affiliations:** 1 Department of Animal Genetics, Breeding and Reproduction, College of Animal Science and Veterinary Medicine, Shenyang Agricultural University, Shenyang 110866, China; 2 Department of Basic Veterinary Medicine, College of Animal Science and Veterinary Medicine, Shenyang Agricultural University, Shenyang 110866, China

**Keywords:** donkey, mammary gland, lactation, integrative interaction, transcriptome

## Abstract

Donkey milk is consumed by humans for its nutritional and therapeutic properties. MicroRNAs (miRNAs) and messenger RNAs (mRNAs) have been implicated in the regulation of milk component synthesis and mammary gland development. However, the regulatory profile of the miRNAs and mRNAs involved in lactation in donkeys is unclear. We performed mRNA-seq and miRNA-seq and constructed coexpression regulatory networks for the mammary glands during the lactating and nonlactating period of jennies. We identified 3144 differentially expressed (DE) mRNAs (987 upregulated mRNAs and 2157 downregulated mRNAs) and 293 DE miRNAs (231 upregulated miRNAs and 62 downregulated miRNAs) in the lactating group compared to the nonlactating group. The DE miRNA target mRNA were significantly associated with pathways related to RNA polymerase, glycosphingolipid biosynthesis, mRNA surveillance, ribosome biogenesis in eukaryotes, glycerophospholipid metabolism, Ras signaling, and the fly hippo signaling pathway. The mRNA–miRNA coregulation analysis showed that novel-m0032-3p, miR-195, miR-26-5p, miR-23-3p, miR-674-3p, and miR-874-3p are key miRNAs that target mRNAs involved in immunity and milk lipid, protein, and vitamin metabolism in the jenny mammary gland. Our results improve the current knowledge of the molecular mechanisms regulating bioactive milk component metabolism in the mammary glands and could be used to improve milk production in donkeys.

## 1. Introduction

Donkeys are an important domesticated species across the world, and the use of dairy products from donkeys has been widely documented [1]. The protein and lactose contents of donkey milk are close to those of human milk, and the total solids and fat contents are lower than those of human and cow milk [2]. Donkey milk shows antimicrobial and anticancer properties, and its consumption and demand are increasing, especially in Italy and China [3,4].

The mammary gland of jennies is characterized by small volumes, and milk is mainly alveolar [5]. The mammary gland is a complex organ, which synthesizes, secretes, stores, and releases milk; these physiological functions constitute “lactation performance”, which is regulated by an unusually high level of postnatal development during puberty and the reproductive cycle [6]. Mammary gland development includes cyclical periods of growth, differentiation, lactation, and regression, which are modulated by the proliferation and apoptosis of mammary epithelial cells [7]. The production of milk is mainly dependent on the metabolic activity and performance of mammary epithelial cells [8]. During the lactation period, the number and secretory activity of mammary epithelial cells increase, decrease gradually, stop, and remodel. In jennies, the milk yield remains constant for 9–10 months of lactation [9,10]. Peak lactation occurs at about 40–60 d from parturition [10], and the highest test-day milk yield ranges from 1.85 kg/d to 3.80 kg/d [11,12]. After lactation, the mammary gland enters the dry (nonlactating) period with the cessation of milk synthesis and secretion, coupled with energy storage and mammary gland cells remodeling to prepare for the next lactation cycle [13].

Mammary gland development and lactation processes are closely associated with various hormones, regulatory factors, and genes [14,15]. With the development of high-throughput sequencing technology, RNA sequencing (mRNA-seq and small RNA-seq) has emerged as a powerful tool to identify and characterize the genes and microRNAs (miRNA) expressed in mammary glands. Mammary gland development, lactation, and involution and the synthesis of milk ingredients are regulated by several genes [16,17]. However, the genes that control lactation in donkeys and the mechanisms regulating their expression are relatively unknown.

miRNAs are a class of small noncoding RNAs (approximately 18–25 nucleotides) that act post-transcriptionally and negatively regulate gene expression by facilitating the degradation or translational repression of target messenger RNAs (mRNAs) by binding to their 3′ untranslated regions [18,19]. Numerous miRNAs modulate the regulation of mammary gland development, lactation, and involution and the synthesis of milk ingredients by regulating gene expression in murine and domestic animals [20,21,22,23]. However, few studies have used a comprehensive approach based on the integrative analysis of miRNA and mRNA expression profiles in the mammary gland during lactating and nonlactating periods in animals including donkeys. Considering the increasing demand for dairy products from donkeys, it is important to understand the molecular regulatory networks involved in the lactation physiology of donkeys.

In this study, we performed an integrative analysis of the miRNA–mRNA expression profiles in the mammary gland tissues of donkeys in the lactating and nonlactating periods to identify the molecular mechanisms involved in lactation. Further, our results could improve our understanding of the post-transcriptional regulation mechanisms of miRNAs and target gene expression in the mammary glands and facilitate strategies to improve lactation production in donkeys.

## 2. Materials and Methods

### 2.1. Ethical Statement

Animals were handled humanely for biological sample collection. The experimental procedures in this study were approved by the Animal Care and Use Committee of Shenyang Agricultural University (approval no. 202001007).

### 2.2. Tissue Sample Collection

Six Chinese Liaoxi jennies (Lvxianyuan Breeding Farm, Fuxin, Liaoning) aged 6.3 ± 1.2 years with a mean live weight of 280.3 ± 20.1 kg and 2.1 ± 0.4 parities were used in this study. The animals were clinically evaluated following the recommended standards of the donkey sanctuary and were diagnosed as healthy [24] and without a history of mastitis. They were housed in a stable with collective paddocks, had free access to food and water, and were bred and managed under the same conditions. Approximately 100 mg of mammary gland tissue was collected by surgical biopsy (general anesthesia by intramuscular injection of xylazine hydrochloride) from the mid-region of the right mammary gland of each jenny during lactation (60 days postpartum; *n* = 3; L group) and the dry period (nonlactating, nonpregnant; *n* = 3; D group). All mammary gland tissue samples were obtained under sterile conditions. After removing the connective and adipose tissue, all samples were washed three times with ice-cold PBS. Part of the tissue was immediately stored in 4% paraformaldehyde for histological examination, and the remaining tissue was snap-frozen in liquid nitrogen and stored at −80 °C for subsequent RNA extraction.

### 2.3. Histological Examination

Blocks of mammary gland tissue were fixed in 4% paraformaldehyde for 48 h and processed and embedded into paraffin blocks using routine procedures [25]. The tissues were embedded in paraffin blocks, and sections of 3–6 μm were cut from each specimen. Oven-dried sections were deparaffinized with xylene, dehydrated through a graded series of ethanol (100%, 95%, and 80% ethanol) and distilled water, and stained with hematoxylin and eosin. Hematoxylin–eosin-stained sections were analyzed using a light microscope (Leica DM4 B, Wetzlar, Germany) equipped with a Leica DFC7000 T digital camera utilizing the Leica Application Suite X imaging software (Leica Biosystems, Wetzlar, Germany).

### 2.4. RNA Extraction, Library Preparation, and Sequencing

Total RNA was extracted from the mammary gland tissue using ice-cold TRIzol reagent (Invitrogen, Carlsbad, CA, USA) following the manufacturer’s instruction.

For RNA-seq library construction, 10 µg of the total RNA extracted was used. mRNA was enriched with magnetic beads containing Oligo(dT). Double-stranded cDNA was synthesized, and short fragments were cleaved. After adaptor ligation, the cDNAs were amplified. During the quality control (QC) steps, the Agilent 2100 Bioanalyzer (Agilent, Santa Clara, CA, USA) and ABI StepOnePlus Real-Time PCR System (Perkin-Elmer Applied Biosystems, Foster City, CA, USA) were used for the quantification and qualification of the sample library. Samples with RNA integrity numbers (RIN) ≥ 7 were subjected to the subsequent analysis. The obtained cDNA libraries were then sequenced using an Illumina HiSeq 2500 sequencer (Illumina, San Diego, CA, USA).

For small RNA library construction, 3 µg of total RNA was used. In brief, RNAs were ligated to a 5′ adaptor and 3′ adaptor and reverse-transcribed by PCR amplification. Subsequently, fragments between 140 bp and 160 bp in length were enriched to generate a cDNA library. The QC steps were performed as described above. The constructed high-quality cDNA libraries were sequenced using the Illumina HiSeq Xten platform (Illumina, San Diego, CA, USA).

### 2.5. mRNA and miRNA Raw Data Analysis

The quality of the raw data was evaluated using the fastp (version 0.18.0) program. Joint-containing, null, and low-quality sequences were removed, and the original readings were filtered to obtain clean reads. Clean reads were aligned to the reference donkey genome, ASM303372v1, and the equine genome database in the National Center for Biotechnology Information GenBank by Bowtie2 (version 2.2.8, Johns Hopkins University, Baltimore, MD, USA), and HISAT2 (version 2.2.4, Johns Hopkins University, Baltimore, MD, USA) software was used to compare the net sequencing to the genome sequence of the donkeys. Transcript abundance estimation was performed using StringTie (version 1.3.1, Johns Hopkins University, Baltimore, USA).

After removing low-quality reads, the remaining sequences (clean reads) were mapped to the donkey genome using the short oligonucleotide alignment program (SOAP) (http://soap.genomics.org.cn) (accessed on 21 December 2021) with a tolerance of one mismatch. The matched sequences were blasted against Rfam database 11.0 (http://www.sanger.ac.uk/software/Rfam) (accessed on 21 December 2021) and NCBI GenBank database 209.0 (http://blast.ncbi.nlm.nih.gov/) (accessed on 21 December 2021) to identify and remove rRNA, scRNA, snoRNA, snRNA, and tRNA sequences. The remaining reads mapped to genomic repeats and known transcripts (exonic and intronic). Clean reads were processed for computational analysis and compared using miRBase 22.0 to obtain known miRNAs. Sequences that were not mapped to any of the conserved miRNAs were further explored to find novel miRNAs using miRDeep2 (version 2.0.0.7, Berlin Institute for Medical Systems Biology, Berlin-Buch 13125, Germany).

### 2.6. Differential Expression Analysis of mRNA and miRNA

The expression levels of all transcripts were estimated as the fragments per kilobase million (FPKM) index. The normalization index simplified the comparison of expressed mRNAs within a sample. Therefore, the FPKM index was used to determine the differentially expressed (DE) mRNAs. The mRNAs that significantly differed between the two groups were identified with two counts based on the methods in R packages including edgeR and DESeq2. A false discovery rate < 0.05 and a fold change (FC) ≥ 2.0 was set as the threshold for DE mRNAs. The correlation and clustering analysis was performed with the R package pheatmap (version 1.0.8, AT&T Bell Laboratories, New York, USA). The screening criteria for differentially expressed miRNAs (DE miRNAs) were as follows: FC ≥ 2.0 and *p*-value < 0.05.

### 2.7. miRNA Target Gene Prediction

Because miRNAs function by interacting with target genes, target gene prediction is essential when studying miRNA function. TargetScan (version 7.0, Whitehead Institute, Cambridge, MA, USA) and MiRanda (version 3.3a, Memorial Sloan-Kettering Cancer Center, New York, NY, USA) software were used to predict potential target genes of the DE miRNAs. The data predicted by both algorithms were combined, and the intersecting elements were included as candidate target genes.

### 2.8. Gene Ontology (GO) and Pathway Analyses

According to the differential gene test results, a functional enrichment analysis was performed on gene ontology (GO) terms in the molecular function (MF), cellular component (CC), and biological process (BP) categories (http://www.geneontology.org/) (accessed on 15 January 2022). Through a comparison with the Kyoto Encyclopedia of Genes and Genomes (KEGG) database (http://www.genome.jp/kegg/) (accessed on 15 January 2022), pathways that were significantly enriched in DE mRNAs were identified. GO and KEGG analyses were performed with DAVID 6.8 (https://david.ncifcrf.gov/) (accessed on 20 January 2022) and KOBAS 3.0 (http://kobas.cbi.pku.edu.cn/genelist/) (accessed on 20 January 2022) using R based on the hypergeometric distribution. The GO and KEGG pathway enrichment statistics were performed using Fisher’s exact test with a cut-off *q* value < 0.05 considered as significant for both GO terms and pathways.

### 2.9. Integrative Analysis of miRNA–mRNA Pairs

Since mRNAs and miRNAs have potential negative regulatory relationships, we assessed the expression correlation between an miRNA and its predicted target gene using the Pearson correlation coefficient (PCC). Subsequently, the negatively coexpressed miRNA–mRNA pairs with PCC < −0.7 and *p* value < 0.05 were screened to construct miRNA–mRNA networks. The key potential regulatory networks of associated miRNAs and mRNAs were visualized using the Cytoscape software (version 3.8.0, Cytoscape Consortium, San Diego, CA, USA).

### 2.10. Quantitative Real-Time PCR Validation of Differentially Expressed mRNAs and miRNAs

The expression of differentially expressed mRNAs and miRNAs was determined using qRT-PCR. Total RNA was extracted from the mammary gland tissues of six jennies at the same period (three of them in a lactating period and the others in a nonlactating period) and used for cDNA synthesis. cDNA was generated from 1 μg of total RNA using the PrimeScript RT reagent Kit (TaKaRa, Tokyo, Japan), and qRT-PCR was performed using SYBR Premix Ex Taq (TaKaRa, Tokyo, Japan). For miRNA detection, reverse transcription followed by qRT-PCR was performed according to the manufacturer’s protocols using the miRNA First Strand cDNA Synthesis (Tailing Reaction; Shenggong, Shanghai, China). The quantification of miRNA was performed with an MiRNA qPCR Kit (SYBR Green Method) (Shenggong, Shanghai, China). Fluorescent quantitative primers were designed with primer5 (Table S1). GAPDH and U6 snRNA were selected as the internal controls. The LightCycler 96 System (Roche, Basel, Switzerland) was applied to qRT-PCR. For each mRNA and miRNA in the two groups, every reaction was performed in triplicate. FCs were determined by the threshold cycle (CT). The FCs of miRNA expression were calculated using the 2^−ΔΔCt^ method [26]. Finally, the relative expression results were compared with the RNA-seq data.

### 2.11. Statistical Analyses

Comparisons of the relative expression values between the two groups in qRT-PCR were analyzed using the independent-samples *t*-test and SPSS 22.0 (SPSS, Inc., Chicago, IL, USA). The results are expressed as means ± standard deviations (SD). Significant differences between the two groups were considered in terms of the associated *p*-value relative to *p* < 0.05 and *p* < 0.01.

## 3. Results

### 3.1. Morphological Structure

**Figure 1:**
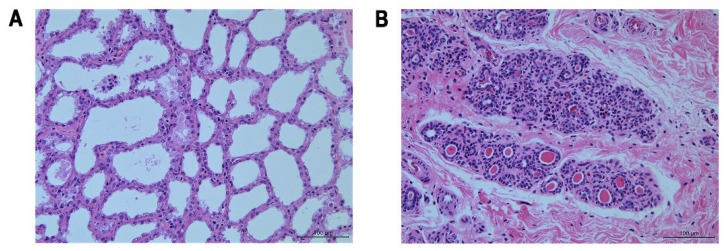
Morphological characteristics of the mammary gland in jennies observed after H & E staining (40×). (**A**) Paraffin section of jenny mammary gland during lactation. (**B**) Paraffin section of jenny mammary gland during the nonlactating period. Nuclei are dyed blue by hematoxylin, and the cytoplasm is stained pink by eosin.

H&E staining showed a variety of cell shapes, large and dilated alveoli containing milk secretion, and a small amount of connective tissue between the alveoli in the lactating mammary glands (Figure 1A). In contrast, small alveoli with narrow lumens lined by small cuboidal cells were observed in the nonlactating mammary glands. In addition, an apparent increase in stromal, connective, and fatty tissue and a thicker alveolar septum were observed in the nonlactating mammary glands (Figure 1B).

### 3.2. Identification of Differentially Expressed mRNAs

**Figure 2:**
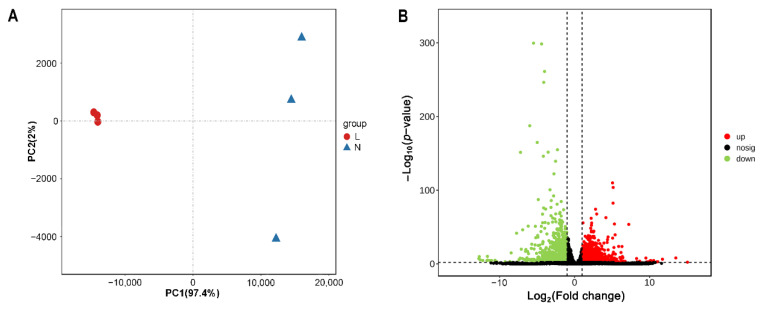
Differential gene expression analysis between L and N groups determined by RNA-seq. (**A**) Principal component analysis (PCA) of differentially expressed (DE) mRNAs. (**B**) Volcano plot of DE mRNAs in jenny mammary glands between L and N groups. The upregulated and downregulated DE mRNAs are indicated by red and green dots, respectively, while the DE mRNAs with no significant difference in the two groups are indicated by black dots. L, lactating mammary glands of jennies; N, nonlactating mammary glands of jennies.

Six cDNA libraries from three jennies in lactation and three jennies in the nonlactating period were sequenced from mammary gland tissues. For each library, clean reads were obtained and ranged from 98.49% to 98.89% after quality filtering. Approximately 90% of the clean reads could be mapped to the donkey reference genome, with a unique match ratio of 86.80–89.58% (Table 1). A principal component analysis (PCA) was performed, which showed that the samples from the L and N groups separated into two distinct clusters (Figure 2A), indicating that the sequencing data qualified for further analysis. A total of 3144 DE mRNAs were identified, of which 987 were upregulated and 2157 were downregulated (|FC| ≥ 2.0, FDR < 0.05) in the lactating group compared to the nonlactating group (Table S2A). A volcano plot was drawn to illustrate significant differences (Figure 2B) according to the FC and FDR values between the two groups. Furthermore, 536 novel mRNAs were identified in the sequencing data.

### 3.3. Identification of Differentially Expressed miRNAs

**Figure 3:**
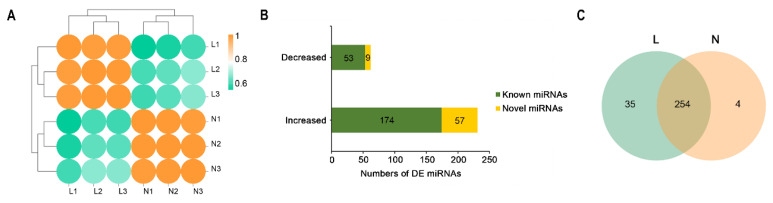
Differential miRNA expression analysis between the L and N groups by miRNA-seq. (**A**) Sample correlation heatmap of miRNAs. (**B**) The histogram shows the number of differentially expressed (DE) miRNAs identified in jenny mammary glands in the L and N groups. (**C**) Venn diagram representation of the common and group-specific DE miRNAs identified in jenny mammary glands of the L and N groups. L, lactating mammary glands of jennies; N, nonlactating mammary glands of jennies.

In the miRNA sequencing data, after removing low-quality reads and sequences shorter than 20 nucleotides and longer than 30 nucleotides in length, 11,161,279–14,222,917 (99.40–99.69% of raw reads) clean reads were obtained (Table 2). Of the clean reads, 62.71%−73.57% of the reads from the two groups were mapped to the reference sequence. The sample correlation heat map from the miRNA expression profiles indicated that three replicate samples from each group had good repeatability (Figure 3A). A total of 293 miRNAs, including 231 upregulated and 62 downregulated miRNAs (|FC| ≥ 2.0 and *p*-value < 0.05), were differentially expressed in the lactating group and nonlactating groups (Table S2B), in which 227 known miRNAs and 66 novel miRNAs were identified (Figure 3B). Among the 293 DE miRNAs, 35 and 4 DE miRNAs were uniquely expressed in the lactating and nonlactating groups, respectively (Figure 3C).

### 3.4. Functional Enrichment Analysis of Differentially Expressed mRNAs

**Figure 4:**
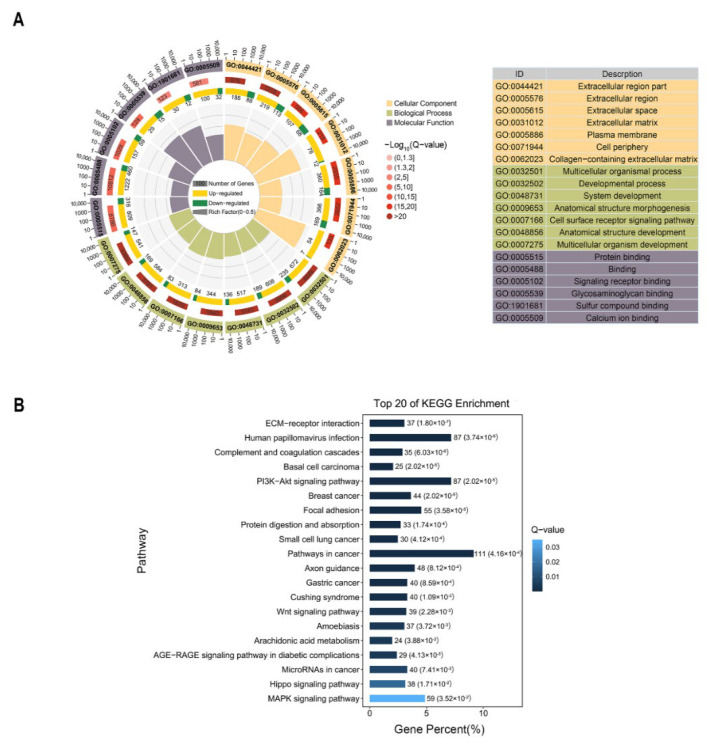
Functional annotation and enrichment analysis for differentially expressed (DE) mRNAs in the mammary glands of the lactating and nonlactating groups. (**A**) GO annotation results of DE mRNAs. The outermost circle shows the items most significantly enriched among the DE mRNAs. The second circle indicates the number of background DE mRNAs and the levels of Q-values. The third circle, consisting of bar graphs, shows the ratio of upregulated DE mRNAs to downregulated ones associated with each GO term, in which yellow represents the upregulated portion and green indicates the downregulated portion. The innermost (fourth) circle indicates the rich factor value for each GO term (the number of DE mRNAs versus the number of non-DE mRNAs associated with the GO term). Each gridline represents 0.1. (**B**) Top 20 significant pathways of KEGG enrichment analysis of DE mRNAs. The ordinate is the pathway, and the abscissa is the enrichment factor.

To improve our understanding of the DE mRNAs in the lactating and nonlactating periods, we performed classification and enrichment analyses using the GO and KEGG pathways of the DE mRNAs. The GO and KEGG enrichment analyses showed that most of the DE mRNAs were mainly involved in biological processes and pathways related to development, morphogenesis, cellular processes, signal transduction, diseases, and metabolism. The 3144 DE mRNAs were classified into three categories through GO enrichment analysis. In the BP category, multicellular organismal processes, developmental processes, system development, anatomical structure morphogenesis, and cell surface receptor signaling pathways were dominantly enriched. The most enriched CCs were in the extracellular category, including the extracellular region, extracellular space, extracellular matrix, and plasma membrane. In the MF category, DE mRNAs were mainly involved in protein binding, signaling receptor binding, glycosaminoglycan binding, and sulfur compound binding (Figure 4A and Table S3A).

The 3144 DE mRNAs were related to 340 KEGG pathways (Table S3B); the top 20 significantly enriched pathways are shown in Figure 4B. The most prevalent pathways associated with DE mRNAs were associated with ECM-receptor interaction, the PI3K-Akt signaling pathway, breast cancer, complement and coagulation cascades, protein digestion and absorption, and arachidonic acid metabolism.

### 3.5. Target Gene Prediction and Functional Enrichment Analysis of DE miRNA

**Figure 5:**
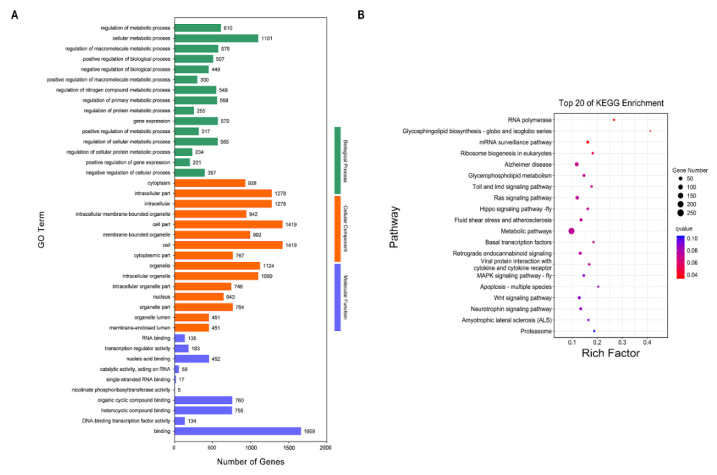
Functional annotation and enrichment analysis for DE miRNA target mRNAs in jenny mammary gland between the lactating and nonlactating groups. (**A**) GO annotation results of differentially expressed (DE) miRNA target mRNAs. The abscissa is the second-level GO term, and the ordinate is the number of DE miRNA target mRNAs in the term. (**B**) Top 20 significant pathways of KEGG enrichment analysis of DE miRNA target mRNAs. The ordinate is the pathway, and the abscissa is the enrichment factor.

A total of 2979 target DE mRNAs corresponding to the 293 DE miRNAs were analyzed (Table S4). Multiple GO terms and pathways were related to mRNAs targeted by the DE miRNAs in the mammary glands obtained from the two groups. Several targeted DE mRNAs were mainly enriched in the regulation of metabolic processes, cellular metabolic processes, and the regulation of macromolecule metabolic processes of BP (Figure 5A, Table S5A). The KEGG pathway analysis revealed that the targeted DE mRNAs were most significantly associated with RNA polymerase, glycosphingolipid biosynthesis, mRNA surveillance, ribosome biogenesis in eukaryotes, glycerophospholipid metabolism, the Ras signaling pathway, and the fly hippo signaling pathway (Figure 5B and Table S5B).

### 3.6. Integrated Analysis of mRNAs and miRNAs

**Figure 6:**
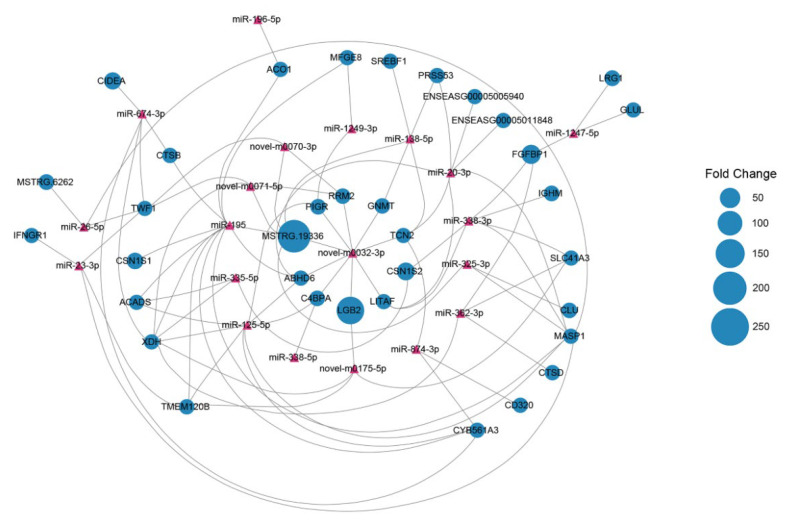
Integrated miRNA-target negative correlation regulatory network. The differentially expressed miRNAs are displayed as red triangles, and the target mRNAs are shown as blue circles. The sizes of blue circles indicate the fold changes in the lactating period compared to the nonlactating period.

To identify potential miRNA target mRNAs involved in immunity and milk lipid, protein, and vitamin metabolism in the jenny mammary gland, the expression profiles of the DE miRNAs and mRNAs were combined for further correlation analysis. We obtained 850 DE mRNAs as putative targets for 293 DE miRNAs through an integrated analysis, presenting a negatively correlated expression pattern (Table S6). The key potential regulatory networks of the miRNA target mRNAs involved in the regulation of immune defense and milk lipid, protein, and vitamin metabolism in the jenny mammary gland were constructed (Table S7). A total of 35 mRNAs were potentially targeted by 20 miRNAs, of which novel m0032-3p, miR-195, miR-26-5p, miR-23-3p, miR-674-3p, and miR-874-3p were key miRNAs, connected by nine, eleven, three, four, five, and three target mRNAs, respectively (Figure 6).

### 3.7. Validation of Differentially Expressed mRNAs and miRNAs by qRT-PCR

**Figure 7:**
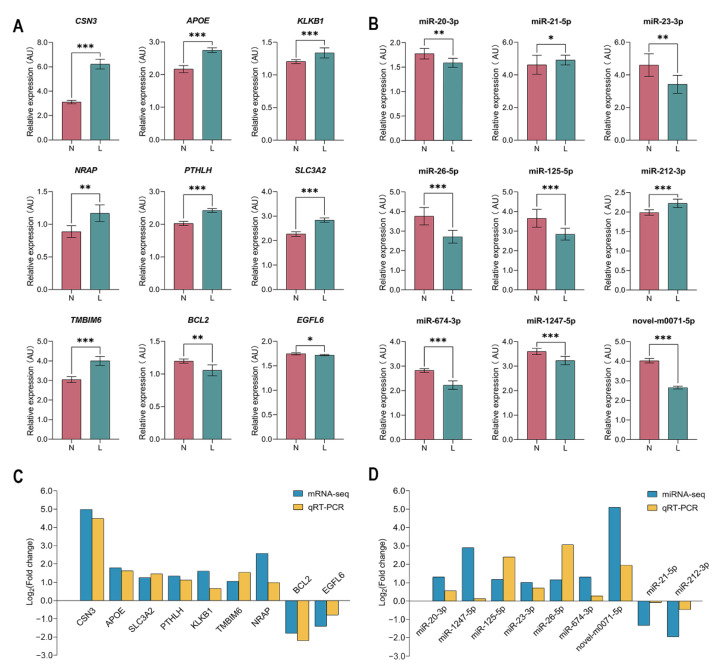
Verification of DE mRNAs and DE miRNAs by qRT-PCR. (**A**) qRT-PCR analysis of nine randomly selected mRNAs. Data represent the means ± SD. (**B**) qRT-PCR analysis of nine randomly selected miRNAs. Data represent the means ± SD. (**C**) Comparison of mRNA expression in terms of Log_2_ (fold change) as assessed by mRNA sequencing and qRT-PCR. (**D**) Comparison of miRNA expression in terms of the Log_2_ (fold change) as assessed by miRNA sequencing and qRT-PCR. L, lactating mammary glands of jennies; N, nonlactating mammary glands of jennies. * *p* < 0.05; ** *p* < 0.01; *** *p* < 0.001.

We randomly selected nine mRNAs and nine miRNAs for qRT-PCR verification. The results showed that the relative expression levels of all the selected mRNAs and miRNAs were significantly different between the two groups (*p* < 0.05; Figure 7A,B). Overall, the expression trends of the selected mRNAs and miRNA obtained from the qRT-PCR data were consistent with that from the sequencing data (Figure 7C,D), indicating high reliability of the sequencing results.

## 4. Discussion

Donkey mammary gland miRNAs and their corresponding target genes have not been identified, and their functionality has therefore not been studied. In this study, we used high-throughput RNA sequencing to determine the interaction patterns of mRNAs and miRNAs in the lactating and nonlactating jenny mammary gland. The miRNA target mRNAs are involved in the regulation of immunity and milk lipid and protein metabolism. To our knowledge, this is the first report of an miRNA–mRNA interaction analysis in lactating and nonlactating jenny mammary gland tissues. Our findings contribute significantly to a better understanding of the molecular regulatory mechanisms governing lactation physiology in donkeys.

Donkey milk has potential antibacterial and immunoprotective effects that could prove useful for protecting vulnerable newborns or promoting adaptive immunity in infants [27,28]. In this study, we identified several immunoprotection-related genes that were upregulated in the lactating jenny mammary gland but not during the nonlactating period. Among these genes, β-lactoglobulin II variant B (*LGB2*; 152.15-FC), immunoglobulin heavy constant mu (*IGHM*; 8.24-FC), polymeric immunoglobulin receptor (*PIGR*; 7.32-FC), MHC class I polypeptide-related sequence B-like (MSTRG.19336; 213.1-FC), zona pellucida sperm-binding protein 3 receptor-like isoform X2 (*C4BPA*; 79.11-FC), and lipopolysaccharide-induced tumor necrosis factor-α (TNF-α) factor (*LITAF*; 2.24-FC) were highly expressed and cotargeted by novel-m0032-3p. *LGB2* encodes a whey protein called β-lactoglobulin II, which is involved in the maintenance of the fetomaternal immune system and shows allergy-preventive as well as allergy-reducing effects [29,30]. *IGHM* is considered an indicator of immunoglobulin mu (IgM). IgM antibodies play a vital role in primary defense mechanisms by recognizing antigens [31]. *PIGR* plays a vital role in immunoglobulin transportation and can transport polymeric immunoglobulins (such as polymeric IgA and pentameric IgM) from the basolateral surface onto the apical surface of epithelial cells by transcytosis [32,33].

A novel gene, MSTRG.19336, was described as major histocompatibility complexes (MHC) class I polypeptide-related sequence B-like. MHC class I is involved in antigen processing and presentation and is upregulated in donkey colostrum whey [34]. It is crucial in protecting newborns from bacterial and other microbial infections [35,36]. In our study, MSTRG.19336 was upregulated 213.1-fold in lactating mammary glands compared with those during the nonlactating period. *C4BPA* is a coregulator of immunity and fat metabolism in bovine mammary epithelial cells and is primarily associated with critical inflammatory and coagulation processes [37]. Further, *LITAF* is involved in the immune response against bacterial and viral infections and can regulate TNF-α transcription, inflammation, proliferation, and apoptosis [38]. Our findings revealed that novel m0032-3p and its targeted genes participated in the innate immune regulation of the jenny mammary gland in lactation and could possibly explain the low prevalence of intramammary infections (i.e., mastitis) in donkeys [39].

Our results showed the significant upregulation of milk protein genes such as α S1 casein isoform X1 (*CSN1S1*; 18.13-FC), α S1 casein isoform X3 (*CSN1S2*; 16.85-FC), cathepsin B (*CTSB*; 4.28-FC), and cathepsin D (*CTSD*; 3.98-FC) in jenny mammary glands during peak-lactation compared to the nonlactating period. Previous studies have identified two types of caseins, αs1-Cn and αs2-Cn, whose encoding genes (*CSN1S1* and *CSN1S2,* respectively) are associated with effects on milk yield, protein, and fat percentages [40]. They have also been related to milk coagulation properties such as rennet coagulation time and curd firmness [41]. *CTSB* and *CTSD* are cathepsin genes involved in the proteolysis of dairy products [42]. Cathepsins are among the principal endogenous proteases and have a significant effect on the physicochemical characteristics and quality of dairy products [43]. In bovine milk, *CTSB* and *CTSD* gene expression increase during lactation [44]. These milk protein genes were predicted to be cotargeted by miR-195, which has anticancer effects and suppresses genes related to cell proliferation, migration, and the invasion of breast cancer cells [45]. Donkey milk can form a weak coagulum under acidic conditions; the high expression of intramammary milk protein genes in lactation suggest that donkey milk could have unique properties suited to the production of yogurt-type products [46].

The synthesis of milk lipid involves multiple complex biological processes and cellular events that are regulated in part by gene expression and affected by miRNAs in the mammary epithelial cells [47]. In this study, twinfilin1 (*TWF1*) was upregulated (2.05-FC) in the lactating jenny mammary glands. *TWF1* is an actin monomer-binding gene that is ubiquitous in eukaryotes from yeast to mammals. It enhances milk triglyceride and casein synthesis and the proliferation of bovine mammary epithelial cells via the mTOR signaling pathway [48]. *TWF1* is predicted to be targeted by putative DE miRNAs, such as miR-26-5p and miR-23-3p. The expression of miR-26 directly regulated genes related to milk triacylglycerol accumulation and unsaturated fatty acid synthesis [49], and the miR-26 family targets members of the PI3K-Akt, MAPK, and fatty acid biosynthesis pathways in goat mammary epithelial cells [50]. miR-23a is involved in the regulation of the mRNA expression of genes associated with milk lipid synthesis in goat mammary gland epithelial cells [51].

*TWF1* was also identified as a predicted target of miR-674-3p, which was significantly upregulated in radiation-induced rat mammary cancer compared to normal mammary tissues [52]. Although the role of miR-674-3p during normal lactation is unclear, the current study showed that it could potentially target four key lipid-metabolism-related genes: *TWF1*, sterol regulatory element binding transcription factor 1 (*SREBF1*; 2.36-FC), cell death-inducing DFFA-like effector A (*CIDEA*; 26.16-FC), and xanthine dehydrogenase (*XDH*; 2.02-FC). *SREBF1* is a key lipogenic transcriptional factor that regulates genes that are involved in milk lipid synthesis [53]. It participates in the AMPK and mTOR signaling pathways that regulate lipid synthesis in bovine mammary epithelial cells [54]. *CIDEA*, a lipid droplet coat gene, plays a positive role in the de novo synthesis and secretion of milk fat. High levels of *CIDEA* are expressed in milk-secreting epithelial cells of lactating murine and bovine mammary tissues [55,56]. In this study, we found a similar trend of high *CIDEA* expression in lactating jenny mammary glands. Another crucial mediator of milk lipid droplet formation, *XDH,* is enriched in the milk fat globule membrane [57]. Compared to that in the nonlactating period, the expression of *XDH* in the mammary glands of dairy cows was significantly upregulated at the onset of lactation [58]. Furthermore, *CIDEA* positively regulated the expression of *SREBF1* and *XDH* in milk lipid accumulation in ruminants [59,60].

miR-874-3p is an important factor in regulating lactogenesis and cell proliferation [61]. Our results revealed that the upregulated genes targeted by miR-874-3p in the lactating jenny mammary gland were transcobalamin 2 (*TCN2*; 2.30-FC), CD320 molecule (3.51-FC), and cytochrome b561 family member A3 (*CYB561A3*; 2.66-FC). These genes are all related to vitamin metabolism and transport. In donkey milk, the B-complex vitamin content is higher than that of human milk, and the vitamin C content is higher than that in dairy cow milk, showing a great similarity with human milk [62]. *TCN2* is essential in the transport of vitamin B12 from the blood to various tissues and organs [63]. *CD320* is expressed in mammary epithelial cells and shows a high affinity for the transcobalamin—vitamin B12 complex [64]. *CD320* regulates transcobalamin degradation in the cell and free vitamin B12 transportation to milk [65]. *CYB561A3*, a member of the CYB561 family, acts as a monodehydroascorbate reductase and is involved in stress defense, iron metabolism, and various neurological processes [66]. These findings indicate that the higher content of B-complex and vitamin C in donkey milk is likely associated with the increased expression of vitamin-related genes in lactating jenny mammary glands.

## 5. Conclusions

To the best of our knowledge, this is the first systematic report on the expression patterns of miRNAs and target mRNAs related to lactation in the mammary glands of jennies. A total of 3144 DE mRNAs and 293 DE miRNAs were identified in the lactating group compared with the nonlactating group. The mRNA–miRNA coregulation analysis showed that the miRNA target mRNAs were mainly involved in immune defense and milk lipid, protein, and vitamin metabolism in the jenny mammary gland. These findings provide a better understanding of the molecular mechanisms regulating bioactive milk component metabolism in the mammary glands and could be used to improve milk production in donkeys.

## Figures and Tables

**Table 1:** Overview of the reads and quality control of the mRNA sequencing libraries from jenny mammary glands.

Sample	Raw Reads	Clean Reads	Clean Reads Ratio	GC (%)	Q20 (%)	Mapped Reads (%)	Unique Reads (%)
L1	52,449,628	51,868,320	98.89	49.86	97.76	89.67	86.80
L2	39,737,124	39,285,118	98.86	49.72	97.95	90.37	87.46
L3	42,857,296	42,258,604	98.60	50.12	98.05	90.04	87.10
N1	40,434,896	39,953,502	98.81	49.00	98.05	91.73	89.58
N2	49,086,804	48,434,360	98.67	49.06	98.05	91.41	89.22
N3	43,879,152	43,217,918	98.49	49.67	98.02	91.39	89.07

**Table 2:** Overview of the reads and quality control of the miRNA sequencing libraries from jenny mammary glands.

Sample	Raw Reads	Clean Reads	Clean Reads Ratio	Mapped Reads	Mapped Reads Ratio	Known miRNA	Novel miRNA
L1	14,446,333	14,222,917	99.45	8,919,639	62.71	710	153
L2	12,710,963	12,510,345	99.40	7,907,070	63.20	720	158
L3	12,474,765	12,322,109	99.48	7,935,039	64.40	718	155
N1	13,844,475	13,694,490	99.69	9,895,214	72.26	702	126
N2	11,290,133	11,161,279	99.68	8,136,513	72.90	682	101
N3	13,671,230	13,486,896	99.68	9,922,828	73.57	697	126

## Data Availability

All data needed to evaluate the conclusions in this paper are present either in the main text or the Supplementary Materials.

## Supplementary Materials

The following supporting information can be downloaded at: https://www.mdpi.com/article/10.3390/genes13091637/s1, Table S1: Primer information of mRNAs and miRNAs used for qRT-PCR; Table S2: Significant differentially expressed (DE) mRNAs were identified between L and N groups (A), significant DE miRNAs were identified between L and N groups (B). L, lactating mammary glands of jennies; N, nonlactating mammary glands of jennies; Table S3: Go enrichment analyses of significant differentially expressed (DE) mRNAs identified between L and N groups (A), KEGG pathway enrichment analysis of significant DE mRNAs identified between L and N groups (B). L, lactating mammary glands of jennies; N, nonlactating mammary glands of jennies; Table S4: Differentially expressed (DE) miRNAs and their target DE mRNAs were identified between L and N groups. L, lactating mammary glands of jennies; N, nonlactating mammary glands of jennies; Table S5: Go enrichment analyses of significant differentially expressed (DE) miRNA target mRNAs identified between L and N groups (A), KEGG pathway enrichment analysis of significant DE miRNA target mRNAs identified between L and N groups (B). L, lactating mammary glands of jennies; N, nonlactating mammary glands of jennies; Table S6: All differentially expressed (DE) miRNA-DE mRNAs target pairs were identified between L and N groups through integrated analysis. L, lactating mammary glands of jennies; N, nonlactating mammary glands of jennies; Table S7: Interaction network of differentially expressed (DE) mRNAs and DE miRNAs.
